# WNT3a and WNT5a Transported by Exosomes Activate WNT Signaling Pathways in Human Cardiac Fibroblasts

**DOI:** 10.3390/ijms20061436

**Published:** 2019-03-21

**Authors:** Edyta Działo, Michał Rudnik, Roman I. Koning, Marcin Czepiel, Karolina Tkacz, Monika Baj-Krzyworzeka, Oliver Distler, Maciej Siedlar, Gabriela Kania, Przemysław Błyszczuk

**Affiliations:** 1 Department of Clinical Immunology, Jagiellonian University Medical College, 30-663 Cracow, Poland; edyta.dzialo@doctoral.uj.edu.pl (E.D.); marcin.czepiel@uj.edu.pl (M.C.); karolina.tkacz@student.uj.edu.pl (K.T.); mibaj@cyf-kr.edu.pl (M.B.-K.); misiedla@cyf-kr.edu.pl (M.S.); 2 Center of Experimental Rheumatology, Department of Rheumatology, University Hospital Zurich, 8952 Schlieren, Switzerland; Michal.Rudnik@usz.ch (M.R.); Oliver.Distler@usz.ch (O.D.); gabriela.kania@uzh.ch (G.K.); 3 Department of Chemical and Cell Biology, Leiden University Medical Center, Leiden University, 2300 Leiden, The Netherlands; R.I.Koning@lumc.nl

**Keywords:** exosomes, WNT3a, WNT5a, cardiac fibroblasts, canonical WNT, non-canonical WNT, cardiovascular diseases

## Abstract

WNT signaling plays an important role in fibrotic processes in the heart. Recently, exosomes have been proposed as novel extracellular transporters for WNT proteins. In this study, we analyzed whether WNT3a and WNT5a carried by exosomes could activate downstream molecular pathways in human cardiac fibroblasts. Exosomes were isolated from conditioned medium of control, WNT3a- and WNT5a-producing L cells by differential ultracentrifugations. Obtained exosomes showed size ranging between 20–150 nm and expressed exosomal markers ALG-2-interacting protein X (ALIX) and CD63. Treatment with WNT3a-rich exosomes inhibited activity of glycogen synthase kinase 3β (GSK3β), induced nuclear translocation of β-catenin, and activated T-cell factor (TCF)/lymphoid enhancer factor (LEF) transcription factors as well as expression of WNT/β-catenin responsive genes in cardiac fibroblasts, but did not coactivate extracellular signal-regulated kinase (ERK), c-Jun N-terminal kinase (JNK), and activator protein 1 (AP-1) signaling pathways. In contrast, exosomes produced by WNT5a-producing L cells failed to activate β-catenin-dependent response, but successfully triggered phosphorylation of ERK1/2 and JNK and stimulated IL-6 production. In conclusion, exosomes containing WNT proteins can functionally contribute to cardiac fibrosis by activating profibrotic WNT pathways on cardiac fibroblasts and may represent a novel mechanism of spreading profibrotic signals in the heart.

## 1. Introduction

Cardiac fibroblasts represent a dominant population of stromal cells in the heart that maintain organ architecture and support its function. During organogenesis and under homeostatic conditions cardiac fibroblasts control extracellular matrix (ECM) turnover by producing ECM components and proteolytic enzymes and their inhibitors [1]. In the injured or failing heart, cardiac fibroblasts play a key role in cardiac tissue remodeling, which often results in fibrotic changes. During fibrogenesis, activation of cardiac fibroblasts promotes excessive accumulation of stromal cells and ECM proteins in the myocardium. Typically, various signaling molecules or mechanical stress activate cardiac fibroblasts for uncontrolled proliferation and ECM overproduction. Most recently, proteins of the WNT family have been implicated in the activation of cardiac fibroblasts in the number of fibrotic cardiac pathologies [2,3,4].

WNTs represent highly conserved secreted glycoproteins encoded in humans by 19 genes, which trigger receptor-mediated signal transduction cascades [5]. Upon binding to the receptor, WNTs can activate various outputs in a β-catenin-dependent (canonical response) or β-catenin-independent (noncanonical response) manner [6]. In the canonical response, WNTs prevent degradation of β-catenin by inhibiting glycogen synthase kinase 3β (GSK3β)-dependent degradation complex. This is followed by translocation of β-catenin into nucleus, where β-catenin activates T-cell factor (TCF)/lymphoid enhancer factor (LEF) transcription factors and regulates expression of WNT/β-catenin target genes [7]. Published data from mouse models specifically pointed to the importance of GSK3β and β-catenin in cardiac fibrosis [8,9].

Noncanonical WNT response is less specific and includes activation of the number of the common downstream signaling cascades, without involvement of β-catenin. For example, noncanonical WNTs have been shown to activate extracellular signal-regulated kinase 1/2 (ERK1/2) and c-Jun N-terminal kinase (JNK) signaling pathways as well as to regulate activity of the downstream activator protein 1 (AP-1) transcription factor [10]. Canonical and noncanonical WNT signaling is triggered by different WNT proteins. WNT3a has been recognized as a potent stimulator of β-catenin-dependent response, while WNT5a was shown to activate β-catenin-independent signaling [11]. In humans, elevated levels of WNT5a have been associated with progressive heart failures [4].

WNT proteins act as intercellular signaling molecules. Transfer of WNT signaling between cells occurs mostly through extracellular space. Most of WNT proteins prior to secretion become modified by lipidation (mainly with palmitate) and glycosylation [12]. Palmitoylated WNTs are hydrophobic and water insoluble, therefore intercellular WNT signaling requires extracellular carriers for WNT proteins. WNT-binding proteins, such as Wntless and Secreted wingless-interacting molecule (Swim), enable secretion of the active WNT complex by binding to lipidated WNTs. Recently, exosomes have been proposed as an alternative transporter of the active WNT proteins [13]. Exosomes represent a subset of extracellular vesicles (EVs) smaller than 150 nm in diameter and are known to transport various cargo components, such as proteins, lipids, and nucleic acids [14]. Virtually all cells actively secrete exosomes in the process of exocytosis. So far, little is known about WNT signalling mediated through exosomes. In this study, we analysed how canonical WNT3a and noncanonical WNT5a transported by exosomes activated WNT-dependent downstream molecular signalling pathways in human cardiac fibroblasts.

## 2. Results

### 2.1. Characteristics of Exosome-Like Extracellular Vesicles Secreted by WNT3a- and WNT5a-Producing L Cells

**Figure 1:**
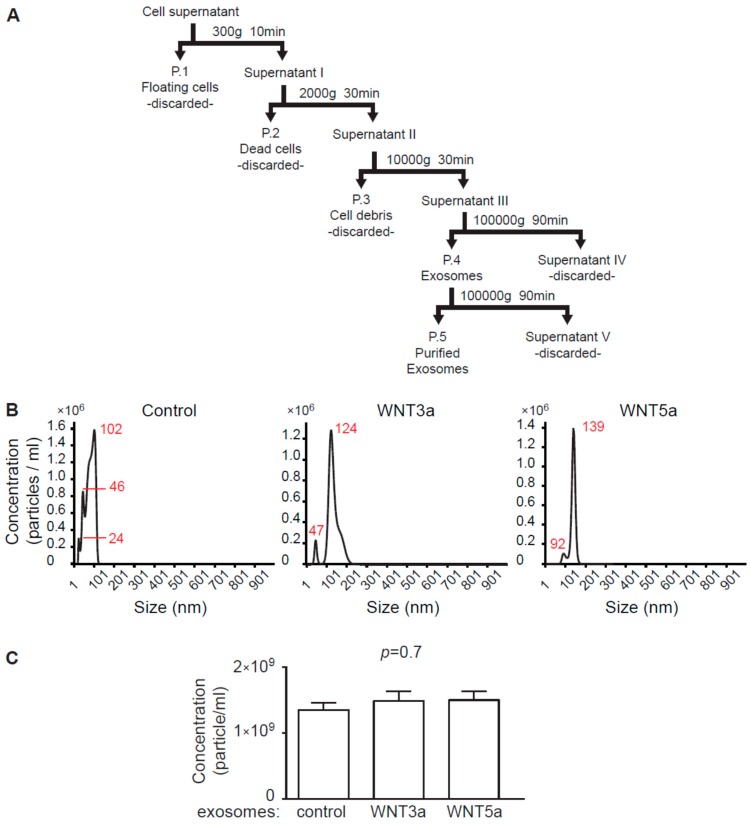
Isolation and nanoparticle tracking analysis (NTA) of exosomes isolated from cell culture supernatants. Panel (**A**) shows a scheme of the exosome isolation protocol. Supernatants of control L, L-WNT3a, and L-WNT5a cell lines cultured for 48 h in exosome-free medium were used to obtain exosomes by serial ultracentrifugations. Panel (**B**) shows representative NTA and panel (**C**) indicates the concentrations of purified exosome stocks (P.5) obtained from the indicated cell lines. Graphs show means ± SEM, *n* = 4, *p* value computed using one-way ANOVA.

**Figure 2:**
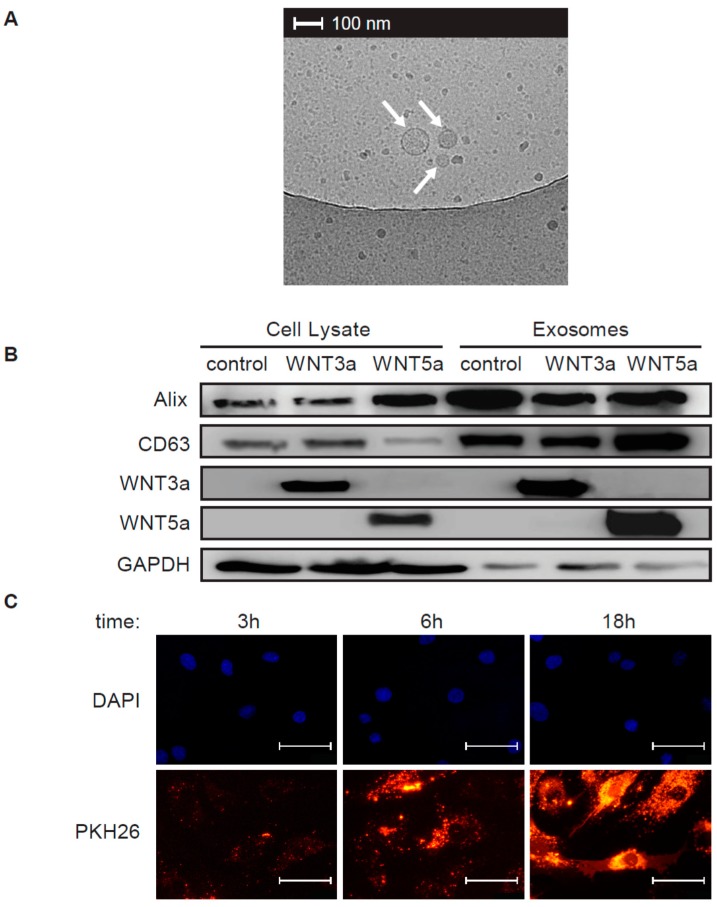
Characteristics of obtained exosomes. Representative transmission electron microscopy (TEM) micrograph showing morphology of exosomes (arrows) is presented in panel (**A**). Immunoblots of exosomal (ALIX, CD63), cytoplasmatic cell (GAPDH) markers, WNT3a and WNT5a proteins in cell lysates and in purified exosomes are presented in panel (**B**). Human cardiac fibroblasts were treated with PKH26-labelled exosomes for 3 h, 6 h, and 18 h. Panel (**C**) shows PKH26-labelled exosomes and cell nuclei (DAPI) at the indicated time point. Bar = 50 μm.

In order to obtain exosomes containing WNT proteins, L-WNT3a, L-WNT5a, and control L cell lines were plated in the exosome-free medium and cultured for 48 h. The use of exosome-free medium allowed to avoid contaminations with bovine exosomes and other EVs, which are commonly present in fetal bovine serum (FBS). To isolate exosomes produced by cells, cell culture supernatants underwent a series of centrifugation and ultracentrifugation steps as presented in the Figure 1A. This procedure resulted in isolation of EVs (P.4—crude exosomes and P.5—purified exosomes) with estimated size ranging between 20–150 nm (Figure 1B). Using this procedure, we obtained 1.2–1.9 × 10^8^ purified exosomes (P.5) from 100 mL cell culture supernatants of L cells. There were no significant differences in the number of produced exosomes among L cell lines (Figure 1C). The size and the round shape of the obtained exosomes were confirmed by transmission electron microscope analysis (Figure 2A).

Exosomes can be distinguished from other types of EVs not only by size, but also by presence of the specific exosomal markers, such as ALG-2-interacting protein X (ALIX) and CD63. Immunoblot analysis showed elevated levels of ALIX and CD63 in purified EVs, while cytoplasmic protein GAPDH was more abundant in cell lysates. Similar results were observed for EVs produced by all three cell lines (Figure 2B). Collectively, our data indicated that obtained EVs showed features typical for exosomes and were further referred to as exosomes.

WNT3a and WNT5a produced by the respective cell line were expected to be loaded into secreted exosomes. Accordingly, immunoblot analysis confirmed presence of WNT3a and WNT5a proteins on exosomal fraction obtained from the respective WNT-producing cell line (Figure 2B). Summarizing, the standard isolation method of exosomes from cell culture supernatants and the use of WNT-producing L cells allowed for successful isolation of exosomes carrying WNT proteins.

### 2.2. Activation of the Canonical WNT/β-catenin Pathway by Exosomal WNTs

**Figure 3:**
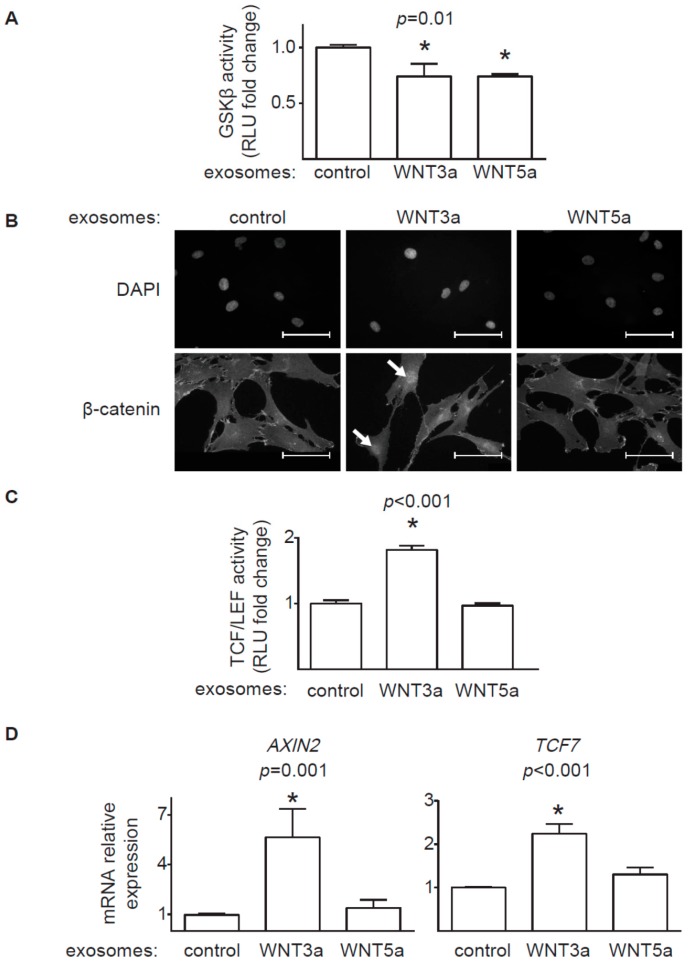
Activation of the canonical WNT/β-catenin pathway in cardiac fibroblasts. Exosomes obtained from L-WNT3a, L-WNT5a, or control L cells were used to stimulate human cardiac fibroblasts. Panel (**A**) shows quantification of GSK3β kinase activity in cardiac fibroblasts 1 h after stimulation with exosomes. Graphs show means ± SEM, *n* = 9, *p* value computed using one-way ANOVA followed by the Dunnett’s multiple comparisons test, * *p* < 0.05 (post-hoc test vs. control). Panel (**B**) demonstrates localization of β-catenin in cardiac fibroblasts 2 h after treatment with exosomes. DAPI stains cell nuclei. Arrows indicate nuclear localization of β-catenin. Data are representative for three independent experiments. Bar = 50 μm. Panel (**C**) shows quantification of TCF/LEF activity in the TCF/LEF-hCF reporter cardiac fibroblasts stimulated with exosomes for 18 h. Graphs show means ± SEM, *n* = 14, *p* values computed using one-way ANOVA followed by the Dunnett’s multiple comparisons test, * *p* < 0.05 (post-hoc test vs. control). Panel (**D**) shows relative mRNA levels of WNT/β-catenin target genes in cardiac fibroblasts treated with exosomes for 24 h. Graphs show means ± SEM, *n* = 7, *p* values computed using the Kruskal–Wallis analysis followed by Dunn’s multiple comparison test, * *p* < 0.05 (post-hoc test vs. control).

In the next step, we analyzed how exosomes secreted by the WNT-producing L cells activated WNT downstream signaling pathways in human cardiac fibroblasts. In order to visualize the interaction of exosomes with target cells, we treated cardiac fibroblasts with exosomes labelled with PKH26 fluorescent dye. As a result, we observed increasing accumulation of exosomes on cardiac fibroblasts over time (Figure 2C). In the canonical response, WNTs induce nuclear translocation of β-catenin and transcription of β-catenin target genes. Stimulation of cardiac fibroblasts with purified exosomes obtained from L-WNT3a or L-WNT5a cells suppressed activity of GSK3β—an enzyme involved in β-catenin degradation. In fact, the inhibitory effect was the same for both WNTs (Figure 3A). Next, we analyzed cellular localization of β-catenin in the target cells after treatment with exosomes. Cardiac fibroblasts treated with control exosomes and WNT5a-rich exosomes showed uniform distribution of β-catenin within analyzed cells, while treatment with exosomes derived from L-WNT3a cells triggered nuclear accumulation of β-catenin (Figure 3B). In the nucleus, β-catenin has been recognized to regulate gene expression by activating TCF/LEF responsive elements. To monitor nuclear activity of the WNT/β-catenin pathway, we introduced TCF/LEF reporter system into cardiac fibroblasts. Stimulation of the TCF/LEF-hCF reporter cardiac fibroblasts with exosomes showed enhanced activity of the TCF/LEF responsive elements only upon stimulation with exosomes obtained from L-WNT3a cells (Figure 3C). Furthermore, we analyzed expression of WNT/β-catenin target genes *AXIN2* and *TCF7* in unmodified cardiac fibroblasts treated with exosomes. In comparison to treatment with control exosomes, cardiac fibroblasts treated with WNT3a-rich, but not with WNT5a-rich exosomes showed elevated levels of *AXIN2* and *TFC7* transcripts (Figure 3D). All these results clearly showed that WNT3a, but not WNT5a carried by exosomes, could effectively activate the WNT/β-catenin pathway in human cardiac fibroblasts.

**Figure 4:**
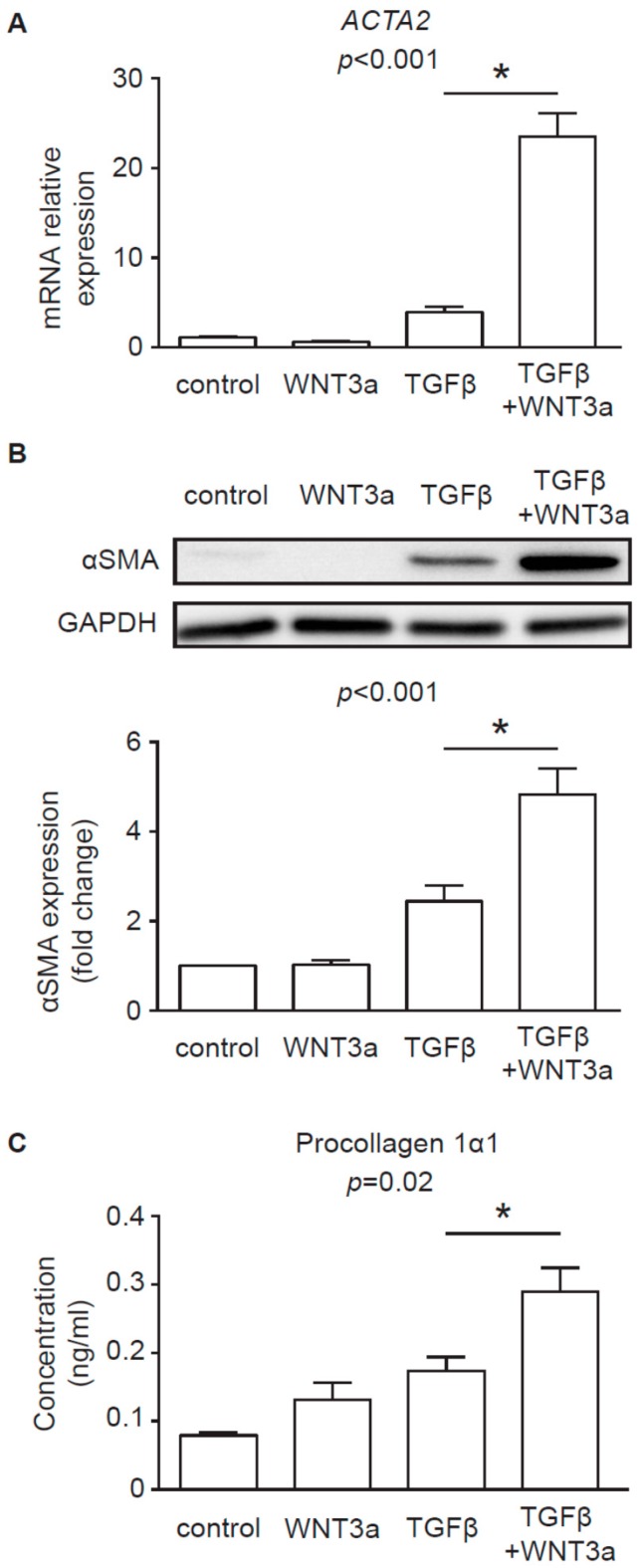
WNT3a promotes myofibroblast differentiation in the presence of TGF-β. Human cardiac fibroblasts were stimulated with conditioned medium produced by L-WNT3a or control L cells in the presence or absence of 10 ng/mL TGF-β. Panel (**A**) indicates expression of *ACTA2* (gene encoding αSMA, *n* = 5) and panel (**B**) illustrates representative immunoblot and the respective quantifications of αSMA (*n* = 5) 72 h after stimulation. Panel (**C**) shows collagen I alpha 1 levels measured in supernatants of cardiac fibroblasts treated for 5 days as indicated (*n* = 4). Graphs show means ± SEM, *p* values computed using two-way ANOVA followed by Tukey’s multiple comparisons test, * *p* < 0.05 (post-hoc TGF-β vs. TGF-β+WNT3a).

To assess the profibrotic properties of WNT3a, cardiac fibroblasts were stimulated with conditioned medium of L-WNT3a and control L cells in the presence or absence of transforming growth factor beta (TGF-β). WNT3a alone failed to upregulate myofibroblast-specific alpha-smooth muscle actin (αSMA), both at the mRNA (Figure 4A) and protein level (Figure 4B), and did not significantly increase type I collagen production (Figure 4C). As expected, stimulation with TGF-β effectively triggered profibrotic changes in cardiac fibroblasts. Costimulation with WNT3a, however, further enhanced TGF-β-induced responses (Figure 4A–C). These data indicated that WNT3a could indeed exaggerate profibrotic signaling in cardiac fibroblasts.

### 2.3. Activation of the Noncanonical WNT Pathway by Exosomal WNTs

**Figure 5:**
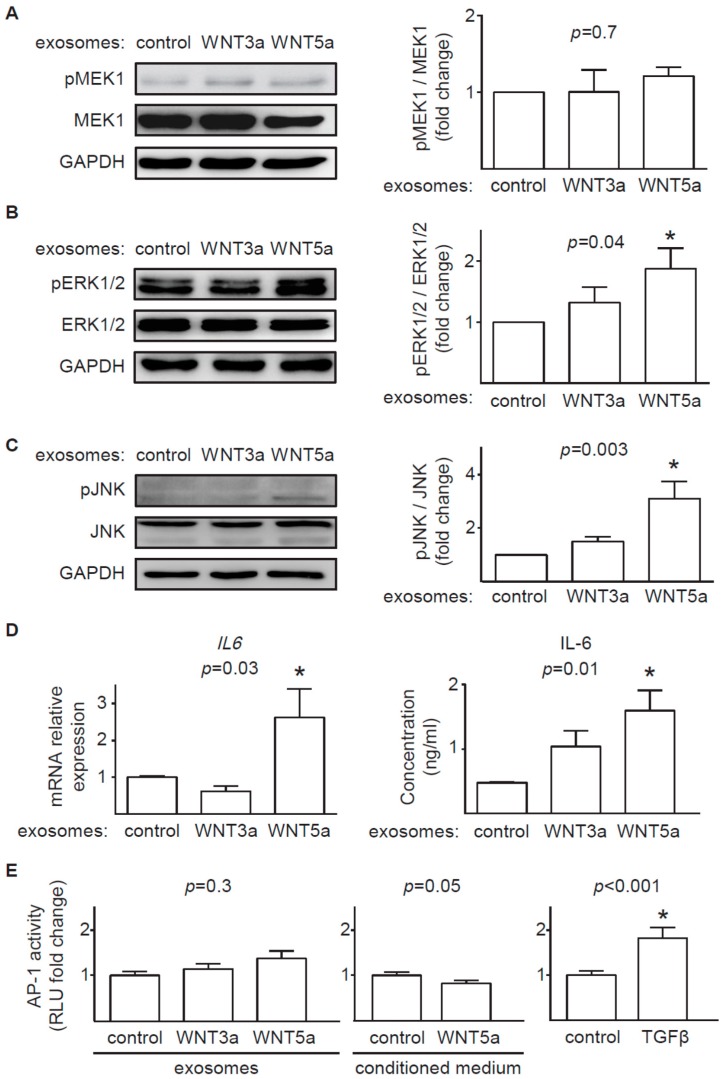
Activation of the noncanonical WNT pathway in cardiac fibroblasts. Human cardiac fibroblasts were stimulated with exosomes produced by L-WNT3a, L-WNT5a, or control L cells. Panels (**A**–**C**) illustrate representative immunoblots and the respective quantifications of pMEK1 to total MEK1 (**A**, *n* = 3), pERK1/2 to total ERK1/2 (**B**, *n* = 6), and pJNK to total JNK (**C**, *n* = 4) 3 h after stimulation of cardiac fibroblasts with the respective exosomes. Graphs show means ± SEM, *p* values computed using the Kruskal–Wallis analysis followed by Dunn’s multiple comparison test. * *p* < 0.05 (post-hoc test vs. control). Panel (**D**) indicates *IL6* gene expression (*n* = 9, left) and IL-6 protein measured in supernatants (*n* = 5, right) of cardiac fibroblasts treated with indicated exosomes for 24 h. Graphs show means ± SEM, *p* values computed using one-way ANOVA followed by Dunnett’s multiple comparisons test, * *p* < 0.05 (post-hoc test vs. control). Panel (**E**) shows quantification of AP-1 activity in AP-1-hCF reporter cardiac fibroblasts after treatment with the respective exosomes (left), conditioned medium (middle) or 10 ng/mL TGF-β for 18 h. Graphs show means ± SEM, *n* = 14, *p* values computed using one-way ANOVA followed by the Dunnett’s multiple comparisons test (for exosomes), * *p* < 0.05 (post-hoc test vs. control) or Student *t*-test.

In addition to β-catenin-dependent signaling, WNTs can activate a number of β-catenin-independent responses in target cells. In this study, we analyzed activation of MEK/ERK and JNK/AP-1 transduction pathways. Treatment with exosomes containing WNT3a or WNT5a proteins showed unaffected phosphorylation of MEK1 in cardiac fibroblasts in comparison to treatment with control exosomes (Figure 5A). Interestingly, WNT5a-rich but not WNT3a-rich exosomes induced phosphorylation of ERK1/2 in cardiac fibroblasts (Figure 5B), suggesting MEK-independent activation of ERK pathway by the noncanonical WNT in our model. Furthermore, exosomes containing WNT5a, but not WNT3a, also triggered phosphorylation of JNK (Figure 5C). In the next step, we analyzed production of profibrotic IL-6. Stimulation with WNT5a-rich exosomes stimulated IL-6 production on transcriptional and protein levels (Figure 5D). Furthermore, we measured activation of the AP-1 reporter system in cardiac fibroblasts. Neither exosomes nor conditioned medium obtained from L-WNT3a or L-WNT5a cells were able to regulate AP-1 activity in AP-1-hCF reporter cardiac fibroblasts (Figure 5E). TGF-β is known to stimulate AP-1 activity. Treatment with TGF-β successfully upregulated AP-1 activity in AP-1-hCF reporter cells (Figure 5E) confirming functionality of the AP-1 reporter system in transduced cardiac fibroblasts. Summarizing, our data suggest that exosomal WNT5a can induce selected noncanonical WNT outputs in human cardiac fibroblasts.

## 3. Discussion

Although WNTs play important roles in cardiac development, in the adult heart under homeostatic conditions, WNT signaling is quiescent. Following heart injury associated with cardiac tissue remodeling, WNT signaling is reactivated and promotes fibrotic changes. Published data from animal models demonstrated that WNT antagonists could effectively reduce development of fibrotic changes in the affected hearts [2,3,15,16]. These data indicated that active WNTs were present in the extracellular space. Recently, exosomes have been proposed as a possible extracellular carrier for WNT proteins [13]. So far, exosomal WNTs have not been reported in fibrotic myocardium, but detailed analysis of WNTs in extracellular space in the heart was not performed yet. On the other hand, data from noncardiac models provided evidence that various cell types could produce exosomes containing functional WNTs [17,18,19].

In this study, we used WNT-producing L cells as sources of exosomal WNTs. In line with previous findings [13,20], we confirmed that WNT-producing L cells secrete WNT3a and WNT5a, at least partially, on exosomes. WNT3a represents an example of canonical WNT, which activates a β-catenin-dependent response. Our data clearly showed that exosomal WNT3a could effectively trigger WNT/β-catenin signaling in human cardiac fibroblasts. We observed that WNT3a-rich exosomes inhibited GSK3β activity. It led to nuclear translocation of β-catenin and transcriptional activation of TCF/LEF responsive elements and expression of WNT/β-catenin target genes *AXIN2* and *TCF7*. Importantly, exosomes carrying canonical WNT3a did not stimulate ERK, JNK, and AP-1 outputs. Thus, our data suggest that exosomal WNT3a specifically activate WNT/β-catenin signaling without coactivation of the noncanonical WNT pathway. Data from mouse models pointed to WNT/β-catenin pathway in cardiac fibroblasts as an important player in myocardial fibrogenesis in vivo. Accordingly, loss of β-catenin in cardiac fibroblasts reduced interstitial fibrosis induced by pressure overload [9]. Furthermore, activation WNT/β-catenin pathway by inhibiting GSK3β in cardiac fibroblasts promoted fibrogenesis in postinfarcted hearts [8]. In line with these findings, our data confirmed that, in the presence of TGF-β, WNT3a could effectively enhance profibrotic response in human cardiac fibroblasts. In the light of these results, it seems that canonical WNT ligands carried on exosomes might potentially exacerbate fibrotic processes in the heart by activating WNT/β-catenin signaling.

In contrast to prominent response of the canonical WNT pathway to exosomal WNT3a, WNT5a-rich exosomes only partially activated analyzed noncanonical WNT pathways in cardiac fibroblasts. We found that exosomal WNT5a inhibited GSK3β activity (but did not trigger the downstream WNT/β-catenin response), activated ERK1/2 and JNK pathways and induced production of profibrotic IL-6. Similar activation of ERK1/2 and IL-6 were observed in mouse cardiac fibroblasts stimulated with recombinant WNT5a [4]. WNT5a delivered on exosomes failed, however, to phosphorylate MEK1 suggesting that WNT5a does not trigger the classical mitogen-activated protein kinase (MAPK) cascade in cardiac fibroblasts. Furthermore, WNT5a did not activate AP-1 transcription factor, suggesting irresponsiveness of a WNT/planar cell polarity (PCP) pathway in the analyzed cells. It has to be noted, that different noncanonical WNT outputs can be activated depending on the cell type. In this study we did not address the effect of exosomal WNTs on activation WNT/Ca^2+^ pathway and regulation of a nuclear factor of activated T cells (NFAT) transcription factor activity, which may play a relevant role in noncanonical WNT signaling in cardiac fibroblasts.

The idea that WNTs can be transported on exosomes has important mechanistic implications. Firstly, as exosomes can transport cargo over longer distances, WNTs produced even in extracardiac tissues might functionally contribute to cardiac fibrosis. So far, however, distribution of extracellular WNTs to exosomal and nonexosomal (associated with WNT-binding proteins) fractions in the injured heart remains unknown. Secondly, systemic spreading of exosomes can activate all cell types in the affected region. Thus, WNTs associated with exosomes would likely affect not only fibroblasts, but also cardiac cells, endothelial cells, and others. It has been demonstrated that sustained activation of β-catenin in endothelial cells causes heart failure [21] and WNTs can inhibit proliferation of cardiac progenitor cells in the infarcted heart [22]. It seems that exosomal WNTs could exert multiple pathogenic effects in the injured heart. Finally, WNT carrier might modulate the effector function of WNTs through interaction with the receptor complex or affecting biding affinity of the WNT ligand to its receptor. More research is needed to elucidate this aspect of WNT biology.

Summarizing, our data provide a potential mechanism on how WNT signaling can spread in fibrotic hearts. We demonstrated that intercellular WNT signaling could occur through exosomal transport. This mode of action could effectively activate the profibrotic WNT/β-catenin signaling pathway and, to some extent, noncanonical WNTs in cardiac fibroblasts. Future studies will reveal how much exosomes contribute to WNT-mediated fibrosis in the injured heart.

## 4. Materials and Methods

### 4.1. Cell Cultures

Primary fetal human cardiac fibroblasts were originally obtained from Cell Applications (Cell Applications, San Diego, USA). L-WNT3a (overexpressing WNT3a), L-WNT5a (overexpressing WNT5a) and L (control line for L-WNT3a and L-WNT5a) cell lines were obtained from ATCC (ATCC, Manassas, USA). Cells were cultured in the Dulbecco’s modification of Eagle medium (DMEM, Corning, New York, USA) supplemented with 10% FBS (EURx, Gdansk, Poland), 1:100 penicillin/streptomycin, 100 mM nonessential amino acids (all Corning, New York, USA) and 50 mM β-mercaptoethanol (Sigma-Aldrich, Taufkirchen, Germany). L-WNT3a and L-WNT5a cell lines were selected with medium containing G418 (Sigma-Aldrich). Human cardiac fibroblasts were cultured up to 12 passages. Cells were passaged using standard protocol with 0.25% trypsin (Corning) and seeded 24 h before experiments. All cells used in this study were mycoplasma-free. Cell culture supernatants from L-, L-WNT3a, and L-WNT5a cultured for 48 h were collected, filtered (pore size 0.2 μm, Carl Roth, Karlsruhe, Germany) and used fresh for cardiac fibroblasts stimulation. 

### 4.2. Exosome Isolation

Exosome isolation procedure followed recent guidelines of the International Society for Extracellular Vesicles [23]. For exosome collection, L-, L-WNT3a, and L-WNT5a cells were cultured in an exosome-free DMEM medium for 48 h. Preparation of the exosome-free medium: to remove bovine exosomes from FBS, DMEM culture medium supplemented with 20% FBS was centrifuged in the Sorvall WX 80+ ultracentrifuge with T-1270 fixed angle rotor (WX Ultra Series, Thermo Fisher Scientific, Waltham, USA) for 7 h at 100,000 g at 4 °C and the supernatant was diluted 1:1 with DMEM and supplemented with 1:100 penicillin/streptomycin, 100 mM nonessential amino acids (all Corning) and 50 mM β-mercaptoethanol (Sigma-Aldrich). Cell culture supernatants were collected and frozen. 100 mL of the cell culture supernatants were subjected to sequential centrifugation steps at 300× g, 2000× g, and 10,000× g before pelleting exosomes at 100,000× g in the ultracentrifuge for 90 min at 4 °C. Pellets containing exosomes were suspended in 500 μL phosphate buffered saline (PBS, Corning), filtered (pore size 0.2 μm, Carl Roth) and centrifuged again for 90 min at 100,000 g at 4 °C. Obtained exosomes were resuspended in 100 μL of exosome-free DMEM culture medium and were used as exosome stock for experiments (Figure 1A). For immunoblotting analysis, pelleted exosomes were resuspended in 100 μL RIPA buffer (25 mM Tris HCl pH 7.6, 150 mM NaCl, 1% NP-40, 1% sodium deoxycholate, 0.1% SDS, Cell Signaling Technology, Beverly, USA). Exosomes obtained from L cells were used as control exosomes.

### 4.3. Exosome Measurements

Exosome size distribution and concentration was determined by nanoparticle tracking analysis (NTA) using a NanoSight system: LM10HS microscope equipped with the LM14 488 nm laser module (Malvern Instruments Ltd., Malvern, UK). Samples were diluted 500× in PBS to provide counts within the detection range of the instrument. One-minute duration videos were recorded for each type exosomes. Particle movement was analyzed with the NTA 3.1 NanoSight software according to the manufacturer’s protocol. The NTA software was optimized to first identify and then track each particle on a frame-by-frame basis.

### 4.4. Transmission Electron Microscopy

3 μL of exosome stock was put on a cupper EM grid (Quantifoil R2/2) that was glow-discharged in air. Excess was blotted for one second using filter paper at 99% humidity and room temperature and plunged into liquid ethane at −183 °C using a plunger (EMGP, Leica, Wetzlar, Germany). Samples were transferred to a Tecnai F20 TEM (Thermo-Fischer Scientific) and images were recorded at 10,000× magnification on a Gatan 2kx2k CCD camera behind a 2001 energy filter operated at 20 keV slit width.

### 4.5. Exosome labelling

100 μL of freshly isolated exosome stocks (P.5) were labeled with PKH26 Red Fluorescent Cell Linker Kit (Sigma-Aldrich) according to manufacturer’s protocol, washed and resuspended in 100 μL exosome-free medium. Human cardiac fibroblasts were treated with 10 μL PKH26 labeled exosomes for 3 h, 6 h or 18 h, washed with PBS and fixed with 4% paraformaldehyde for 5 min. DAPI (ThermoFisher Scientific) was used to label nuclei. Immunofluorescence was analyzed using Olympus BX53 microscope equipped with Olympus XC50 camera (Olympus, Tokyo, Japan).

### 4.6. Generation of TCF/LEF and AP-1 Reporter Cardiac Fibroblasts

The pGreenFire-1-TCF/LEF plasmid expressing luciferase under control of TCF/LEF response elements and pGF-AP1-mCMV-CMV-EF1α-Puro plasmid expressing luciferase under control of AP-1 transcriptional response elements were purchased from System Biosciences (System Biosciences, Palo Alto, USA). Lentiviral particles were produced in HEK293T cells (ATCC) by cotransfection with packaging plasmids pMD2.G and psPAX2 (Addgene, Watertown, USA, plasmids #12259 and #12260 gifted by Dilder Trono) according to the Addgene protocol. Lentiviruses were harvested 72 h after transfection, centrifuged and filtered through 0.45 μm membrane filters (VWR International, Radnor, USA). The biological titer was determined by flow cytometry of transduced human cardiac fibroblasts as described elsewhere [24]. For lentiviral transduction, 2.5 × 10^3^ cardiac fibroblasts were plated on 96-well plates and transduced with lentiviral particles for 72h. Lentiviral transfection was performed using a multiplicity of infection (MOI) of 1. Human cardiac fibroblasts transduced with the pGreenFire-1-TCF/LEF plasmid were further called TCF/LEF-hCF and transduced with the pGF-AP1-mCMV-CMV-EF1α-Puro plasmid were further called AP-1-hCF reporter cardiac fibroblasts.

### 4.7. Luciferase Assay

To measure the activity of the TCF/LEF and AP-1 responsive elements in cardiac fibroblasts, TCF/LEF-hCF and AP1-hCF reporter cardiac fibroblasts were stimulated with 5 μL of the exosome stock diluted in 100 μL culture medium, 100 μL conditioned medium or 10 ng/mL human recombinant TGF-β (Peprotech, Rocky Hill, USA) for 24h. Luciferase expression in the reporter cells was assessed using the Bright-Glo Luciferase Assay System (Promega, Madison, USA). Bioluminescence was measured by SyneryHT plate reader (BioTek, Winooski, USA) or Wallac Victor2 1420 Multilabel counter. (Perkin-Elmer, Waltham, USA).

### 4.8. Western Blotting

Cardiac fibroblasts were stimulated with 25 μL of the exosome stock diluted in 1.5 mL culture medium for 1 h or 3 h or with conditioned medium of L-WNT3a or control L cells in the presence or absence of 10 ng/mL TGF-β for 72 h. Total cellular proteins were extracted using RIPA buffer supplemented with protease and phosphatase inhibitors (Thermo Fisher Scientific). Freshly isolated exosomes were directly resuspended in RIPA buffer supplemented with protease and phosphatase inhibitors. Equal amounts of protein content (25 μg protein of cell lysates and exosomes for L cells and 15–20 μg of cell lysates for cardiac fibroblasts) were loaded into 12% polyacrylamide gels, resolved by SDS-PAGE, and transferred to polyvinylidene difluoride membranes (Bio-Rad, Hercules, USA). The membranes were blocked with 5% bovine serum albumin (BSA) and 0.05% Tween 20 in PBS for 1 h. The membranes were incubated with primary antibodies at 4 °C overnight followed by washing and 1 h incubation at room temperature with horseradish peroxidase (HRP)-conjugated secondary antibodies. Protein abundance was detected using a Western Blotting Substrate (Thermo Fisher Scientific) and imaged by using a ChemiDoc instrument (Bio-Rad). Results were analyzed with ImageJ software (NIH, Bethesda, USA). The following antibodies were used: rabbit anti-CD63 (1:500, Bio-Rad), rabbit anti-GAPDH (1:5000), mouse anti-ALIX (1:1000), rabbit anti-WNT3a (1:1000), rabbit anti-WNT5a/b (1:1000), rabbit anti-αSMA (1:1000, Biolegend, San Diego, USA), rabbit anti-MEK1 (1:2000), rabbit anti-phospho-MEK1 (1:1000), rabbit anti-ERK1/2 (1:1000), rabbit anti-phospho-ERK1/2 (1:1000), rabbit anti-SAPK/JNK (called further JNK, 1:1000) and rabbit anti-phospho-SAPK/JNK (called further p-JNK, 1:1000, all Cell Signaling Technology).

### 4.9. Immunoprecipitation and GSK3β-Activity Assay

Cardiac fibroblasts were stimulated with 25 μL of the exosome stock diluted in 1.5 mL medium for 1h. Total cellular proteins were extracted using the lysis buffer (1 M Tris- HCl pH 7.5, Triton X-100, Sucrose, 0.5 M EDTA, 10 mM EGTA, 1.5 mM β-mercaptoethanol) supplemented with protease and phosphatase inhibitors (Thermo Fisher Scientific). For immunoprecipitation equal amount of cell lysates were incubated with anti-GSK3β antibody (12 μg/mL, BD Biosciences, Heidelberg, Germany) for 1h at 4 °C, followed by incubation with protein G-Sepharose beads (Invitrogen, Carlsbad USA) diluted 2-fold in kinase reaction buffer (50 mM Tris-HCl, pH 7.5, 150 mM NaCl, 0.03% Brij-35 and 0.1% β-mercaptoethanol, 10 mM MgCl) at 4 °C for 1 h. The activity of the GSK3β immune complexes were analyzed with the ADP-Glo™ Kinase Assay (Promega) according to manufacturer’s protocol.

### 4.10. Immunofluorescence

For immunofluorescence, cardiac fibroblasts were cultured on gelatin-coated cover slips and stimulated with 10 μL of the exosome stock diluted in 500 μL medium for 2 h. Prior incubation with antibodies, cells were fixed with 4% paraformaldehyde for 5 min at room temperature, permeabilized with 0.01% Triton X-100 for 5 min, and blocked with 5% BSA in PBS for 20 min. Specimens were incubated with mouse anti-β-catenin (1:500, ThermoFisher Scientific) antibody for 1 h, washed, and incubated with AlexaFluor555 anti-mouse IgG secondary antibody for 45 min (1:1000, ThermoFisher Scientific). DAPI (ThermoFisher Scientific) was used to label nuclei. Immunofluorescence was analyzed using Leica DM5500 B fluorescence microscope with DFC365 FX camera (Leica Microsystems).

### 4.11. Quantitative RT-PCR

For mRNA analysis cardiac fibroblasts were stimulated with 10 μL of the exosome stock diluted in 500 μL medium for 24 h. Alternatively, cells were stimulated with conditioned medium of L-WNT3a or control L cells in the presence or absence of 10 ng/mL TGF-β for 72h. Total RNA was extracted with Trizol Reagent (Invitrogen). 200 ng total RNA was used to synthesize complementary DNA using NG dART RT Kit (EURx). Quantitative real-time PCR was performed using SYBR Green PCR Master Mix (EURx) and oligonucleotides complementary to transcripts of the analyzed genes using the Quant Studio 7 Real-Time PCR system (Applied Biosystems, Foster City, USA). The following oligonucleotides were used in this study: *AXIN2*: 5′-TAACCCCTCAGAGCGATGGA-3′ and 5′-CCTCCTCTCTTTTACAGCAGGG-3′, *TCF7*: 5′-CCAAGAATCCACCACAGGAGG-3′ and 5′-GCCTAGAGCACTGTCATCGG-3′, *ACTA2*: 5′-GACAATGGCTCTGGGCTCTGTAA-3′ and 5′-ATGCCATGTTCTATCGGGTACTT-3′, *IL6*: 5′-GGCACTGGCAGAAAACAACC-3′ and 5′-CACCAGGCAAGTCTCCTCAT-3′, *GAPDH*: 5′-GGGAAGCTTGTCATCAATGGA-3′ and 5′-TCTCGCTCCTGGAAGATGGT-3′. Transcript levels of *GAPDH* were used as endogenous reference and relative gene expression was calculated using the 2^−ΔΔ*C*t^ method.

### 4.12. ELISA

Type I collagen was measured in cell culture supernatants using Human Procollagen I alpha 1 DuoSet ELISA (R&D Systems, Minneapolis, USA) and IL-6 was measured in cell culture supernatants using IL-6 Human Uncoated ELISA Kit (Thermo Fisher Scientific) according to the manufacturer’s protocol.

### 4.13. Raw Data and Statistics

Experimental results are presented as mean ± SEM. Raw data are available in the Supplementary Materials. Normally distributed and data with homogenous variance (Brown–Forsythe test) were compared using a Student’s *t*-test, one-way ANOVA followed by Dunn’s post-hoc test or two-way ANOVA followed by Tukey’s post-hoc test for the indicated comparisons. Other data were analyzed using a Kruskal–Wallis test followed by Dunn’s test of multiple comparisons. All analyses were computed using GraphPad Prism 6 software. All data are presented as mean ± SEM. Differences were considered as statistically significant for *p* < 0.05.

## Supplementary Materials

Supplementary materials can be found at https://www.mdpi.com/1422-0067/20/6/1436/s1.
