# Supplementary material for: WNT3a and WNT5a Transported by Exosomes Activate WNT Signaling Pathways in Human Cardiac Fibroblasts
Source: Int J Mol Sci. 2019 Mar 21;20(6):1436. doi: 10.3390/ijms20061436 (PMC6472055; doi:10.3390/ijms20061436)

Supplementary Materials

Działo *et al.* WNT3a and WNT5a Transported by Exosomes Activate WNT Signaling Pathways in Human Cardiac Fibroblasts

Supplementary Table 1 Raw data of Figure 1C- exosome stocks concentrations [particles/ml]

Sample	control	WNT3a	WNT5a
1	1.62E+09	1.42E+09	1.79E+09
2	1.43E+09	1.89E+09	1.62E+09
3	1.16E+09	1.45E+09	1.43E+09
4	1.20E+09	1.20E+09	1.16E+09

Supplementary Table 2 Raw data of Figure 3A - GSKβ activity [relative RLU]

Sample	control	WNT3a	WNT5a
1	1.153	0.965	0.668
2	0.935	0.733	0.713
3	0.966	0.901	0.764
4	0.941	0.101	0.709
5	1.052	0.922	0.716
6	0.974	1.032	0.744
7	1.016	0.910	0.865
8	0.958	0.758	0.748
9	1.037	0.193	0.738

Supplementary Table 3 Raw data of Figure 3C - TCF/LEF activity [relative RLU]

Sample	control	WNT3a	WNT5a
1	0.908	1.761	0.973
2	0.936	1.326	0.927
3	1.094	1.511	1.001
4	1.057	1.539	1.085
5	1.029	1.873	0.936
6	1.038	1.743	1.001
7	0.816	1.845	0.992
8	1.122	2.039	0.927
9	0.743	1.944	1.070
10	1.078	1.925	1.152
11	1.179	1.921	0.893
12	1.433	1.981	1.214
13	0.751	1.469	0.938
14	0.765	1.500	0.660

Supplementary Table 4 Raw data of Figure 3D - *AXIN2* mRNA [relative expression]

Sample	control	WNT3a	WNT5a
1	0.596	1.437	2.908
2	1.188	10.377	3.512
3	0.863	9.223	0.261
4	0.764	4.685	0.554
5	1.343	2.949	0.584
6	0.983	3.401	1.236
7	1.077	16.012	0.726

Supplementary Table 5 Raw data of Figure 3D - *TCF7* mRNA [relative expression]

Sample	control	WNT3a	WNT5a
1	0.999	1.623	1.336
2	1.013	1.664	2.458
3	0.989	1.689	1.212
4	0.985	3.420	1.403
5	1.015	3.231	1.183
6	0.926	2.286	0.657
7	1.023	1.868	1.132

Supplementary Table 6 Raw data of Figure 4A – *ACTA2* mRNA [relative expression]

Sample	Control	WNT3a	TGFβ	WNT3a+TGFβ
1	1	0.45	2.26	20.52
2	0.93	0.39	3.02	19.39
3	1	1.03	4.56	33.54
4	1	0.63	4.66	20.62
5	1.6	0.53	5.22	23.64

Supplementary Table 7 Raw data of Figure 4C – Procollagen Iα1 ELISA [µg/ml]

Sample	Control	WNT3a	TGFβ	WNT3a+TGFβ
1	0.08	0.09	0.18	0.28
2	0.08	0.08	0.22	0.23
3	0.07	0.18	0.13	0.39
4	0.09	0.16	0.16	0.26

Supplementary Table 6 Raw data of Figure 5D - *IL6* mRNA [relative expression]

Sample	control	WNT3a	WNT5a
1	0.898	0.823	1.706
2	1.102	0.966	7.083
3	0.937	0.778	4.366
4	1.096	0.961	5.206
5	1.031	0.278	1.206
6	0.970	1.297	1.135
7	1.045	0.132	0.856
8	0.871	0.190	0.953
9	1.117	0.127	1.109

Supplementary Table 7 Raw data of Figure 5D - IL-6 ELISA [ng/ml]

Sample	control	WNT3a	WNT5a
1	0.496	1.350	1.001
2	0.452	1.425	1.009
3	0.490	1.513	1.298
4	0.513	0.290	2.554
5	0.425	0.615	2.116

Supplementary Table 8 Raw data of Figure 5E (exosome stimulation) - AP1 activity [relative RLU]

Sample	control	WNT3a	WNT5a
1	0.655	0.648	0.650
2	1.700	1.257	0.808
3	0.646	0.708	0.515
4	1.056	0.785	1.181
5	1.892	1.014	0.762
6	0.907	0.913	0.452
7	0.873	1.252	0.956
8	0.645	0.332	0.926
9	0.627	0.559	2.951
10	0.937	1.155	1.723
11	1.136	1.278	1.818
12	1.022	1.846	1.960
13	0.937	1.524	1.893

14	1.146	1.893	1.685

Supplementary Table 9 Raw data of Figure 5E (conditioned medium stimulation) - AP1 activity [relative RLU]

Sample	control	WNT5a
1	1.216	0.899
2	0.954	0.777
3	0.830	0.791
4	1.108	0.662
5	0.893	0.677
6	0.912	0.491
7	1.590	0.534
8	0.619	0.431
9	0.879	0.358
10	0.956	1.104
11	0.718	0.942
12	0.960	1.220
13	1.835	1.211
14	0.852	0.951

Supplementary Table 10 Raw data of Figure 5E (TGFβ stimulation) - AP1 activity [relative RLU]

Sample	control	TGFβ
1	1.216	1.928
2	0.954	1.817
3	0.830	1.282
4	1.108	2.827
5	0.893	1.899
6	0.912	1.431
7	1.590	1.595
8	0.619	2.932
9	0.879	0.661
10	0.911	1.441
11	1.510	1.521
12	0.891	2.613
13	0.771	0.816
14	0.990	1.713

Supplementary Figure 1 Original Immunoblots of αSMA

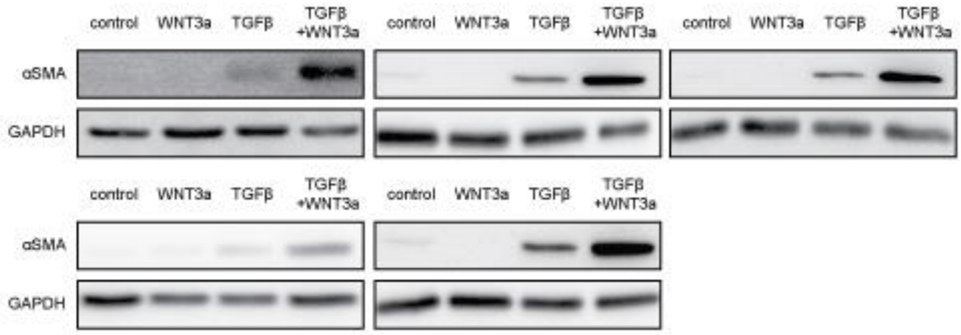

Supplementary Figure 2 Original Immunoblots of (A) pMEK and MEK1. (B) pERK and ERK. (C) pJNK and JNK

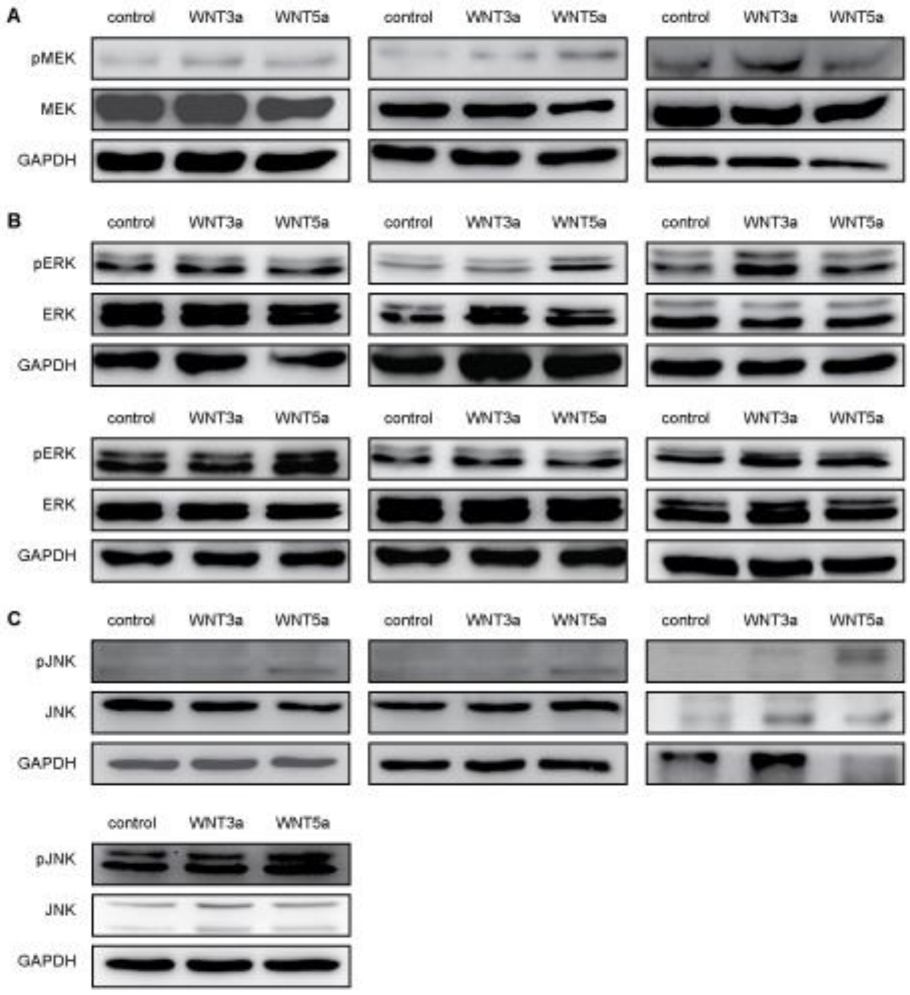

Supplement: Supplementary file 1 [file ijms-20-01436-s001.pdf]
